# Supplementary material for: Micron-Sized Silica-PNIPAM Core-Shell Microgels with Tunable Shell-To-Core Ratio
Source: Gels. 2022 Aug 18;8(8):516. doi: 10.3390/gels8080516 (PMC9407347; doi:10.3390/gels8080516)
Supplement: Supplementary file 1 [file gels-08-00516-s001.zip › kuk-etal-SI-revised.pdf]

Supplementary Information

Micron-sized silica-PNIPAM core-shell microgels with tunable shell-to-core ratio

*Keumkyung Kuk [1], Lukas Gregel [1], Vahan Abgarjan [1], Caspar Croonenbrock [1], Sebastian Hänsch [2], and Matthias Karg [1]**

[1]Institut für Physikalische Chemie I: Kolloide und Nanooptik, Heinrich-Heine-Universität Düsseldorf, Universitätsstr. 1, 40225 Düsseldorf, Germany

[2]Center for Advanced Imaging, Heinrich Heine University Düsseldorf, Universitätsstr. 1, 40225 Düsseldorf, Germany

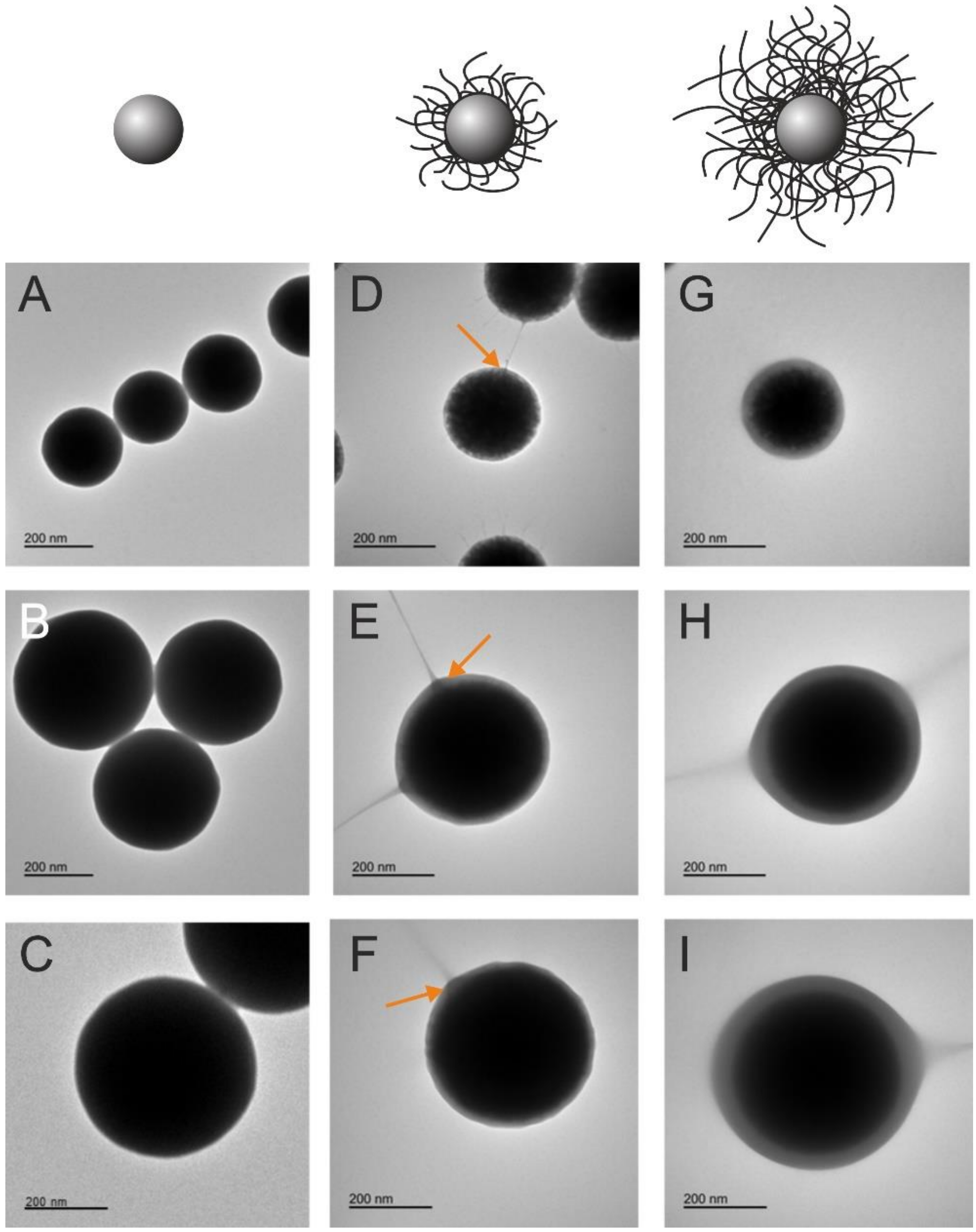

Figure S1. Higher magnification TEM images of CS microgels with variously sized cores: C_{245} (A), C_{388} (B), and C_{455} (C), with thin shells: $C_{245}S_{1.68}$ (D), $C_{388}S_{2.12}$ (E), $C_{455}S_{2.11}$ (F) and with thicker shells: $C_{245}S_{2.89}$ (G), $C_{388}S_{2.62}$ (G), $C_{455}S_{2.34}$ (G).

Influencing parameters in CS microgel synthesis at low TSC. A surfactant, such as SDS, can be used in the synthesis to prevent the aggregation of the cores but it also influences the final size of the CS microgels. For classical microgels, the effect of surfactant is well known: It stabilises the forming primary microgels via charge repulsion thereby driving the systems closer to good-solvent conditions. The smaller the primary microgels are, the higher is the final

number of microgels, which consequently leads to smaller final size of microgels for a given amount of monomer in the system and higher homogeneity. [1-3] For CS microgels synthesized via seeded precipitation polymerization, however, the number of seeds is predetermined by the added number of cores and thus the same mechanism cannot hold. We found that CS microgels nonetheless follow the same trend as the classical microgels. **Figure S2A** shows swelling curves of CS microgels synthesized with various concentrations of SDS: 0 mM ($C_{433}S_{1.99}$-0, light green), 0.2 mM ($C_{433}S_{1.75}$-$SDS_{0.2}$, green), 2 mM ($C_{433}S_{1.63}$-SDS_{2}, dark green). Apart from the influence of SDS, we also investigated the role of the initiator (KPS) concentration and polymerization temperature, which has a similar influence on the size of the CS microgels, as shown in **Figure S2B**. Light blue represents 0.01 wt% KPS ($C_{433}S_{1.99}$-0), blue 0.03 wt% ($C_{433}S_{1.73}$-$KPS_{0.03}$), dark blue 0.05 wt% ($C_{433}S_{1.61}$-$KPS_{0.05}$), respectively.

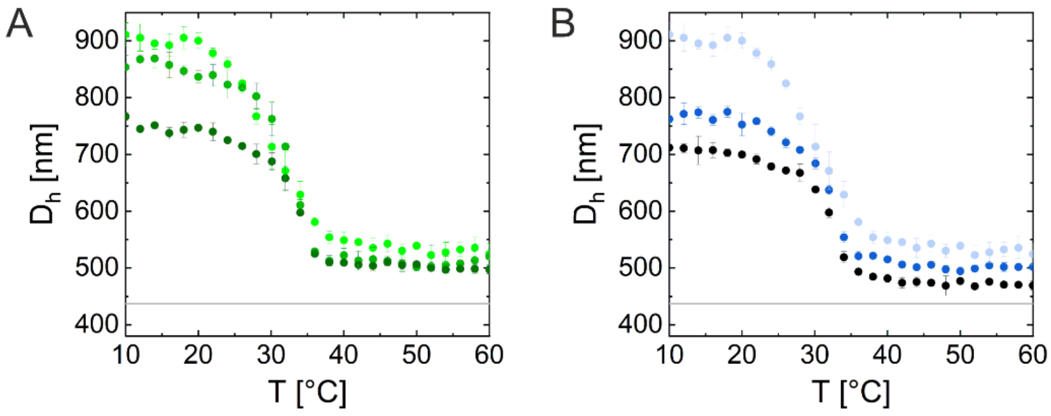

Figure S2. A) Swelling curves of CS microgels synthesized with increasing SDS concentrations (light green: 0 mM, green: 0.2 mM, dark green: 2 mM, grey line: core diameter by TEM. B) with increasing KPS concentrations (light blue: 0.01 wt%, blue: 0.03 wt%, dark blue: 0.05 wt%, grey line: core diameter by TEM.

The synthesis temperature is proven to exert even stronger influence on size of the microgels. For instance, over 2.5 μm sized classical microgels can be synthesized via precipitation polymerization with temperature ramp, usually at a lower temperature range. [4, 5] In contrast to the effect of surfactant or initiator, the lower temperature reduces the number of primary

microgels and therefore leads to the larger microgels. **Figure S3A** shows the influence of the synthesis temperature on the size of the CS microgels. The empty pink stars, filled light red circles, red squares, and dark red triangles represent the CS microgels synthesized with a temperature ramp (45 - 65 °C, $C_{340}S_{3.36}$-T_{45-65}) and at fixed temperatures of 60 °C ($C_{340}S_{3.00}$-T_{60}), 70 °C ($C_{340}S_{2.86}$-T_{70}), and 80 °C ($C_{340}S_{2.72}$-T_{80}), respectively. As for the classical microgels, the size of CS microgels also increased with decreasing synthesis temperature. The CS microgel synthesized via temperature ramping, meanwhile, did not show a strong increase in size and exhibited considerably higher polydispersity when assembled in 2D. Additionally, more efficient stirring also seemed to improve the efficiency in PNIPAM shell growth. **Figure S3B** shows PNIPAM shell encapsulation at low TSC (0.005 g/ml) at three different synthesis temperatures: 60 °C, 70 °C, 80 °C. The filled symbols represent the syntheses stirred with a KPG stirrer with a moon-shaped stirrer blade, and the empty symbols with the egg-shaped magnetic stirring bar. The synthesis at 60 °C stirred with egg-shaped magnetic stirring bar stopped stirring an hour after the initiation due to macroscopic aggregates around the stirring bar.

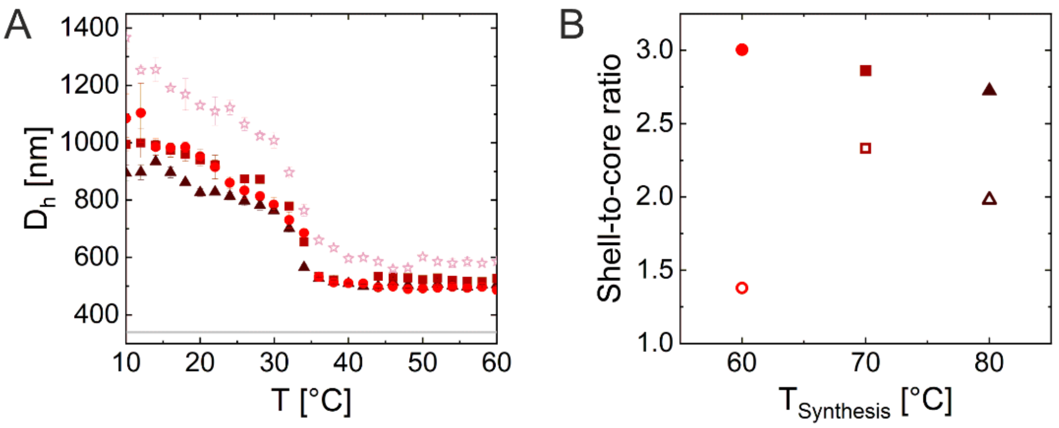

Figure S3. A) Swelling curves of CS microgels synthesized with decreasing temperature (empty light pink stars: 45 - 65°C filled light red circles: 60°C, red squares: 70°C, dark red triangles: 80°C, grey line: core diameter by TEM. B) Comparison between stirring by egg-

shaped magnetic stirring bar (empty scatters) and KPG stirrer with moon-shaped stirrer blades (filled scatters) at various temperature: 60 °C (circles), 70 °C (squares), 80 °C (triangles).

Monolayer preparation. Monolayers of different CS microgels were prepared using interface-mediated self-assembly using a crystallizing dish filled with water. The air/water interface was cleaned by using an aspirator with a tip, as illustrated in **Figure S4A**. Surface pressures were measured using a Wilhelmy film balance. The CS microgels were deposited directly at the air/water interface by injection from ethanolic dispersion using a micropipette. Three different surface pressures (10, 20 and 30 mN/m) were targeted to vary the interparticle distance of the transferred monolayer. The injection was done slowly at a shallow angle while the tip was gently touching the interface until the target surface pressure was reached (**Figure S4B)**. The prepared glass slide was pushed into the water bulk phase through the monolayer close to the edge of the crystallizing dish and moved to the centre. The monolayer was taken from the centre at a steep angle and immediately heat-treated by a heat gun from the bottom side while horizontally held until completely dried (**Figure S4C)**. The bottom side was then carefully wiped with ethanol-soaked tissue.

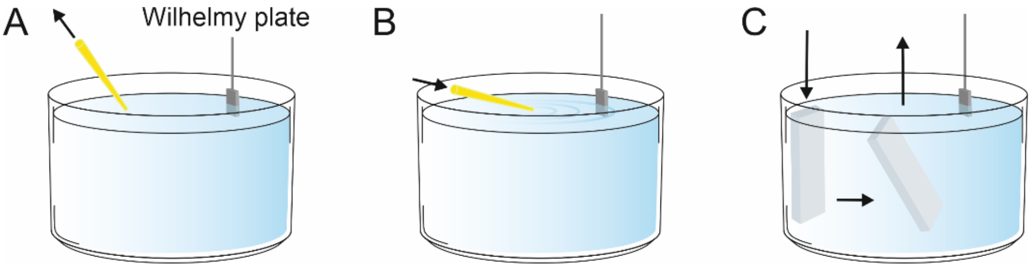

Figure S4. A) Air/water interface cleaning by an aspirator. B) Monolayer deposition with a micropipette at the cleaned air/water interface. C) Monolayer transfer on to the RCA cleaned glass.

Estimation of Area fraction for 2D assembly. Approximately 60,000 particles were counted per sample for the image analysis. The positions of the microgels and number of particles in the probed area (microscopic images) were found using ImageJ and the $D_{c\text{-}c}$ was

obtained from the Gaussian fitting of the first peak of the radial distribution function. **Figure S5** shows the linear relation between $D_{c\text{-}c}^2$ and the area per particle (A/P). Note that the estimated A_f is likely to be underestimated due to the fact that the microgels at the edge of the probed area are often not considered in the image analysis.

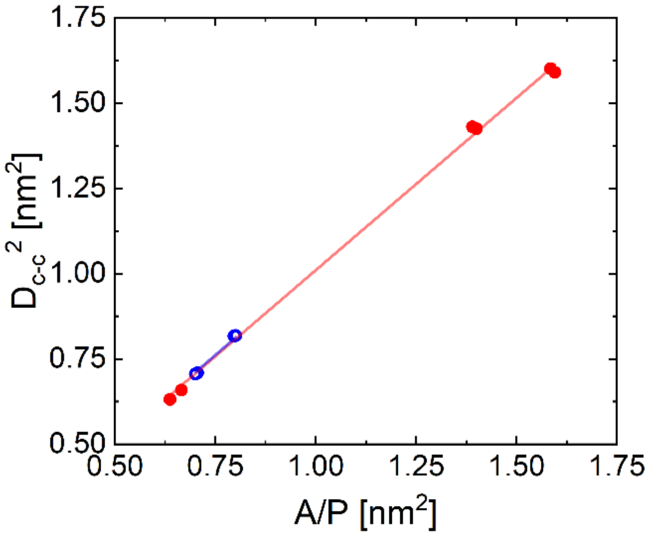

Figure S5. The linear fit for the calculation of A_f from measured data: $C_{245}S_{2.89}$ (empty blue) and $C_{455}S_{2.34}$ (filled red).

Table S1. Chemicals used for the synthesis of silica cores presented in this paper.

	Temp. [°C]	Initial			Added		D_{TEM} [nm]	Dye
		Ethanol [ml]	Water [ml]	Ammonia aq. [ml]	TEOS [ml]	Ethanol [ml]		
C_{245}	40	100	0	10	5	0	245±18	-
C_{388}	50	57	0	19	15	30	388±35	-
C_{455}	60	90	0	20	30	10	455±27	-
C_{340}	60	80	5	25	10	30	340±20	Rhodamine B
C_{433}	50	56	16	28	10	40	433±15	Rhodamine B

The silica particles were synthesised via Stöber procedure. All particles were MPS modified according to the previously published protocol [6] and C_{340} and C_{433} were dyed with Rhodamine B.

Table S2. Chemicals and synthesis parameters used for the preparation of CS microgels presented in this work.

	Core				Core - shell						
**Name**	**D_{TEM} [nm]**	**TSC [g/ml]**	**added [ml]**	**H_2O [ml]**	**NIPAM [g]**	**BIS [g]**	**KPS [g]**	**SDS [g]**	**Temp. [°C]**	**δ 20°C**	**δ 50°C**
*$C_{433}S_{1.99}$-0	437	0.53	0.02	6	0.020	0.001	0.001	0.0000	80	1.99	0.00
*$C_{433}S_{1.75}$-$SDS_{0.2}$	437	0.53	0.02	6	0.020	0.001	0.001	0.0003	80	1.75	1.10
*$C_{433}S_{1.63}$-SDS_{2}	437	0.53	0.02	6	0.020	0.001	0.001	0.0035	80	1.63	1.11
*$C_{433}S_{1.73}$-$KPS_{0.03}$	437	0.53	0.02	6	0.020	0.001	0.002	0.0000	80	1.99	1.13
*$C_{433}S_{1.61}$-$KPS_{0.05}$	437	0.53	0.02	6	0.020	0.001	0.003	0.0000	80	1.83	1.18
$C_{340}S_{2.72}$-T_{80}	340	0.11	2.00	255	1.002	0.072	0.026	0.0000	80	2.72	1.50
$C_{340}S_{2.86}$-T_{70}	340	0.11	2.00	255	1.002	0.072	0.026	0.0000	70	2.86	1.54
$C_{340}S_{3.00}$-T_{60}	340	0.11	2.00	255	1.000	0.072	0.026	0.0000	60	3.00	1.52
$C_{340}S_{3.36}$-$T_{45\text{-}65}$	340	0.11	1.00	255	1.000	0.071	0.026	0.0000	45-65	3.36	1.85
$C_{245}S_{1.26}$-C1	245	0.10	2.00	55	0.101	0.008	0.006	0.0319	80	1.26	1.17
$C_{245}S_{1.35}$-C2	245	0.10	2.00	55	0.202	0.015	0.006	0.0314	80	1.35	1.19
$C_{245}S_{1.44}$-C3	245	0.10	2.00	55	0.302	0.022	0.006	0.0316	80	1.44	1.21
$C_{245}S_{1.43}$-C4	245	0.10	2.00	55	0.401	0.029	0.006	0.0314	80	1.43	1.14
$C_{245}S_{1.45}$-C5	245	0.10	2.00	55	0.500	0.036	0.006	0.0314	80	1.45	1.15
$C_{245}S_{1.52}$-C6	245	0.10	2.00	55	0.600	0.043	0.006	0.0315	80	1.52	1.19
$C_{245}S_{1.57}$-C7	245	0.10	2.00	55	1.000	0.072	0.006	0.0320	80	1.57	1.23
$C_{245}S_{1.85}$-C8	245	0.10	2.00	55	2.001	0.144	0.006	0.0322	80	1.85	1.39
$C_{245}S_{1.68}$	245	0.11	0.33	125	0.076	0.006	0.013	0.0000	70	1.68	1.23
$C_{245}S_{1.71}$	245	0.11	0.33	125	0.088	0.006	0.013	0.0000	70	1.71	1.25
$C_{245}S_{2.07}$	245	0.11	0.33	125	0.095	0.006	0.013	0.0000	70	2.07	1.32
$C_{245}S_{2.21}$	245	0.11	0.33	125	0.100	0.007	0.013	0.0000	70	2.21	1.30
$C_{245}S_{2.49}$	245	0.11	0.33	125	0.106	0.008	0.013	0.0000	70	2.49	1.37
$C_{245}S_{2.89}$	245	0.11	0.33	125	0.265	0.018	0.013	0.0000	70	2.89	1.74
$C_{245}S_{3.07}$	245	0.11	0.33	125	0.404	0.027	0.013	0.0000	70	3.07	1.82
$C_{388}S_{1.75}$	388	0.37	1.00	125	0.192	0.014	0.013	0.0000	70	1.75	1.26
$C_{388}S_{2.12}$	388	0.37	1.00	125	0.304	0.021	0.013	0.0000	70	2.12	1.38
$C_{388}S_{2.43}$	388	0.37	1.00	125	0.383	0.027	0.013	0.0000	70	2.43	1.42
$C_{388}S_{2.62}$	388	0.37	1.00	125	0.502	0.035	0.013	0.0000	70	2.62	1.51
$C_{455}S_{1.25}$	455	0.20	0.36	125	0.086	0.006	0.013	0.0000	70	1.25	1.11
$C_{455}S_{2.11}$	455	0.20	0.36	125	0.229	0.016	0.013	0.0000	70	2.11	1.32
$C_{455}S_{2.23}$	455	0.20	0.36	125	0.502	0.037	0.013	0.0000	70	2.23	1.46
$C_{455}S_{2.30}$	455	0.20	0.36	125	0.601	0.041	0.013	0.0000	70	2.30	1.45
$C_{455}S_{2.25}$	455	0.20	0.36	125	0.751	0.052	0.013	0.0000	70	2.25	1.51
$C_{455}S_{2.34}$	455	0.20	0.36	125	1.003	0.070	0.013	0.0000	70	2.34	1.41

***Small scale synthesis**: See the next paragraph below along with Figure S6 for more details.

Small scale synthesis. 3 - 6 batches were synthesized at a time in 12 ml round bottom glass centrifuge tubes. The tubes were held by a 3D printed holder at around 45° angle, as depicted

in **Figure S6**. The reaction mixtures were prepared in stock, purged with argon for 1 hour and heated up to 80°C while stirring with a winged magnetic stirrer bar. The polymerization was allowed to proceed for at least 3 hours with a constant flow of argon. The dispersions were purified by repeated centrifugation and re-dispersion in water without filtration.

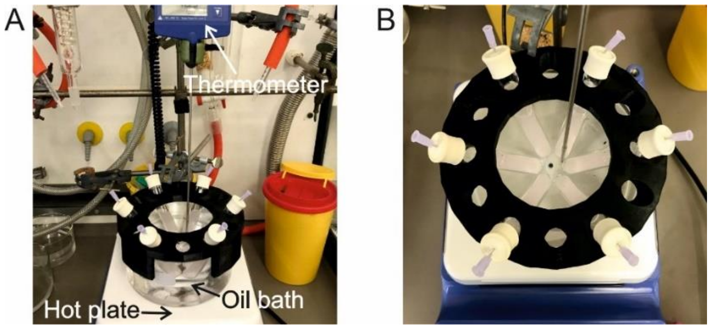

Figure S6. Small scale synthesis setup. A) Side view of the setup. B) Top view.

Small-angle light scattering (SALS). Laser diffraction patterns were recorded by a self-built setup, see **Figure S7**. A blue laser (MediaLas, LDM-20-405, 20 mW, 405 nm) was used as a light source. The diameter of the laser was reduced to 1.19 mm by a beam expander, which consists of two lenses, a pinhole and an iris (Thorlabs). The sample was placed on 3D printed holders. A CCD camera (Thorlabs, DCU223C-MVL6WA) and a paper screen as a detector, the images were captured in dark. The primary beam blocked by a 3D printed beam stop during measurement. The pixel-to-mm ratio was calibrated by using millimetre paper on the paper screen.

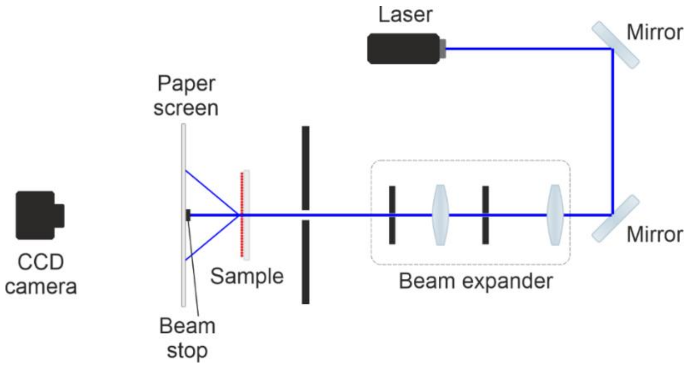

Figure S7. Schematic depiction of the SALS setup that was used to record diffraction patterns from monolayer samples.

1. Wu, X., et al., *The kinetics of poly(N-isopropylacrylamide) microgel latex formation.* Colloid & Polymer Science, 1994. **272**(4): p. 467-477.
2. Andersson, M. and S.L. Maunu, *Structural studies of poly(N-isopropylacrylamide) microgels: Effect of SDS surfactant concentration in the microgel synthesis.* Journal of Polymer Science Part B: Polymer Physics, 2006. **44**(23): p. 3305-3314.
3. Andersson, M. and S.L. Maunu, *Volume phase transition and structure of poly(N-isopropylacrylamide) microgels studied with 1H-NMR spectroscopy in D2O.* Colloid and Polymer Science, 2006. **285**(3): p. 293-303.
4. Meng, Z., M.H. Smith, and L.A. Lyon, *Temperature-programmed synthesis of micron-sized multi-responsive microgels.* Colloid and Polymer Science, 2009. **287**(3): p. 277-285.
5. Still, T., et al., *Synthesis of micrometer-size poly(N-isopropylacrylamide) microgel particles with homogeneous crosslinker density and diameter control.* Journal of Colloid and Interface Science, 2013. **405**: p. 96-102.
6. Rauh, A., et al., *Compression of hard core–soft shell nanoparticles at liquid–liquid interfaces: influence of the shell thickness.* Soft Matter, 2017. **13**(1): p. 158-169.
